# Outcomes for COVID-19 Patients Undergoing Tracheostomy With or Without Extracorporeal Membrane Oxygenation (ECMO)

**DOI:** 10.7759/cureus.55750

**Published:** 2024-03-07

**Authors:** Karim Asi, Daniel Gorelik, Tariq Syed, Apurva Thekdi, Yin Yiu

**Affiliations:** 1 Department of Otorhinolaryngology - Head and Neck Surgery, McGovern Medical School at UTHealth Houston, Houston, USA; 2 Texas Voice Center, Department of Otolaryngology - Head and Neck Surgery, Houston Methodist Hospital, Houston, USA

**Keywords:** surgical airway, ards, tracheostomy, covid-19, ecmo

## Abstract

Introduction

The coronavirus disease 2019 (COVID-19) pandemic led to the more common use of venovenous (VV) extracorporeal membrane oxygenation (ECMO) for adults with acute respiratory distress syndrome (ARDS). While tracheostomy is generally understood to decrease the risks of prolonged endotracheal intubation, there is conflicting data regarding the benefit of tracheostomy in patients on ECMO. The purpose of this study is to determine whether ECMO cannulation before tracheostomy impacted patient outcomes.

Methods

This is a retrospective chart review of patients who underwent tracheostomy for COVID-19-related ARDS at a tertiary academic center from March 2020 through March 2022. Patients were separated into two groups based on whether they were cannulated for ECMO prior to tracheostomy. Fisher’s exact test or Wilcoxon rank sum test was used to compare the two groups.

Results

A total of 24 patients were included in the study, with 13 in the ECMO group and 11 in the non-ECMO group. There was no significant difference in age, comorbidities, race, or gender between the groups. Patients on ECMO had a longer time from admission to intubation (seven days vs. three days, p=.002), were more likely to have multiple intubations (54% vs 9%, p= .033), had increased rates of postoperative bleeding (62% vs. 18%, p = .047), and had a higher mortality rate (39% vs. 0%, p= .041).

Conclusions

ECMO cannulation prior to tracheostomy for COVID-19-related ARDS is associated with poorer outcomes. It is unclear whether this is related to a more severe disease burden in these patients. Further study is needed to evaluate this and guide future management.

## Introduction

The coronavirus disease 2019 (COVID-19) pandemic challenged contemporary patient care principles as the virus’ high transmissibility obliged healthcare workers to modify pre-existing protocols to protect themselves from infection while confronting a novel and deadly pathogen. In the early stages of the pandemic, many COVID-19 patients suffered severe disease and developed acute respiratory distress syndrome (ARDS) requiring intubation and prolonged mechanical ventilation. Early tracheostomy placement for prolonged intubation is generally understood to be beneficial for decreasing rates of ventilator-associated pneumonia, allowing earlier weaning from ventilatory support, and preventing airway complications such as subglottic stenosis [1]. However, there were high levels of trepidation to perform tracheostomy in these patients due to concern for aerosolization and viral transmission to healthcare workers, especially when personal protective equipment (PPE) was in short supply. This apprehension was compounded by limited and conflicting data regarding the benefit of early tracheostomy in COVID-19 patients. While some studies demonstrated decreased length of stay (LOS) and fewer complications in patients undergoing early tracheostomy for COVID-19-related pneumonia, others, including a large systematic review, meta-analysis, and meta-regression, have shown limited benefit of early versus late tracheostomy in terms of mortality and secondary outcomes [2-5].

Over the course of the pandemic, clinicians and researchers have attempted to clarify the understanding of the safety of performing procedures like tracheostomy in COVID-19 patients. Several studies have outlined modified methods for performing open and percutaneous tracheostomy, with minimal risk to surgeons and staff, by utilizing appropriate PPE and techniques to minimize aerosol generation [6,7]. Favier et al. utilized a swine model to evaluate particle aerosolization during open surgical and percutaneous dilatational tracheostomy modified with ventilatory pauses during key portions of the procedure [8]. In the 10 procedures performed, they determined that while major aerosolization did not occur, there were minor leaks from technical issues such as cuff perforation that precluded a completely airtight technique. Berges et al. described a method of using a heat moisture exchanger and surgical mask to cover the tracheostomy tube after placement to decrease aerosolization during coughing and suctioning events as a means of preventing viral transmission to health care workers providing routine tracheostomy care [9]. Further, a virological assessment conducted by Wolfel et al., in the early stages of the pandemic, predicted little risk of infectivity beyond 10 days of symptom onset [10]. Thus, prolonged delay of procedures is unlikely to improve healthcare professional safety and may lead to increased patient morbidity.

With this in mind, an international, multidisciplinary guideline published by McGrath et al. in May 2020 recommended performing tracheostomy after 10 days of mechanical ventilation utilizing enhanced PPE protocols [11]. However, a 2021 comparison of perioperative care protocols from 26 countries revealed that over 90% of protocols recommended delaying tracheostomy beyond 14 days of intubation [12]. This was exemplified by the Canadian Society of Otolaryngology-Head & Neck Surgery (CSO-HNS) task force recommendation in 2021, which recommended delaying tracheostomy until after 20 days of symptom onset and 14 days of intubation. The CSO-HNS also recommended against waiting for negative COVID-19 testing to proceed with tracheostomy and supported the safety of both open and percutaneous approaches to tracheostomy for patients and healthcare personnel when performed after these timeframes [13]. Given data supporting the overall safety of performing and caring for tracheostomies for patients with COVID-19-related ARDS, coupled with the potential benefit of early tracheostomy for these patients, the current study seeks to add to the growing body of literature evaluating the outcomes of patients undergoing open tracheostomy for COVID-19-related ARDS. 

In addition, venovenous (VV)-extracorporeal membrane oxygenation (ECMO) became a widespread treatment method for severe ARDS associated with COVID-19, with nearly 14,000 patients receiving ECMO support from the beginning of the pandemic through August 2022 [14]. Mahmood et al. published a multidisciplinary, multicenter retrospective study on tracheostomy outcomes for patients with COVID-19, including 30 patients who underwent tracheostomy while on ECMO. Of the 30 patients, 25 (83%) could be weaned from ECMO. Those patients had a median of 26 days (range, 23-31 days) from intubation to ECMO weaning and a median of five days (range, 3-8 days) from tracheostomy to ECMO weaning [15]. Importantly, data directly comparing ECMO and non-ECMO patient outcomes was not reported. With regards to the timing of tracheostomy for patients on ECMO, there are conflicting reports surrounding the benefit of early tracheostomy, in part due to an overall poor prognosis. One study of 131 patients who were cannulated for VV-ECMO demonstrated better outcomes for earlier ECMO cannulation and earlier tracheostomy placement [16]. However, another series showed that patients who were on ECMO suffered higher rates of hemorrhagic complications after undergoing early tracheostomy and did not achieve better outcomes [17]. Therefore, they recommended waiting to proceed with tracheostomy until after patients were weaned from ECMO.

These conflicting studies highlight a gap in current knowledge of the optimal protocol and timing of tracheostomy placement, both for COVID-19 patients as a whole and for those on ECMO support. Our tertiary center had a relatively high number of COVID-19 patients cannulated for VV-ECMO, many of whom underwent open tracheostomy placement. This gave the opportunity to expand the current understanding of the use of ECMO and tracheostomy placement as adjunct treatments for ARDS related to COVID-19. Furthermore, this adds to the scientific foundation that will guide the management of future respiratory viral epidemics. The aim of this study is to analyze the effect of ECMO cannulation on the outcomes of patients who underwent tracheostomy for COVID-19-related ARDS.

This article was previously presented as a meeting abstract at the American Broncho-Esophagological Association (ABEA) annual meeting at Combined Otolaryngology Spring Meetings (COSM) in Boston on May 3-7, 2023.

## Materials and methods

Study design

This was a retrospective, case-control study conducted at a tertiary academic center, the Texas Voice Center in the Department of Otolaryngology at Houston Methodist Hospital, Houston, Texas, United States, to examine the effect of ECMO cannulation on the outcomes of patients who underwent tracheostomy for COVID-19-related ARDS. The study was approved by the Houston Methodist Research Institute Institutional Review Board (approval number: PRO00035517). The patient list was compiled based on Current Procedural Terminology coding related to tracheostomy placement (CPT 31600).

Inclusion and exclusion criteria

All patients who underwent tracheostomy for COVID-19 ARDS by two fellowship-trained laryngologists at the Texas Voice Center in the Department of Otolaryngology at Houston Methodist Hospital from March 2020 through March 2022 were identified and included. Patients were excluded if they did not have a positive COVID-19 test prior to tracheostomy, if they were under 18 years old, and if records were incomplete with regards to hospital course and outcomes.

Data collection

Charts were reviewed for demographic and baseline characteristic data in addition to information about hospital course and outcomes. Patients were subsequently separated into two cohorts based on whether they were cannulated for ECMO prior to tracheostomy placement.

Statistical analysis

To assess the difference in outcomes based on ECMO status, Fisher’s exact test was performed for categorical data and Wilcoxon rank sum test was used to analyze continuous data. Statistical significance was set at P < 0.05. 

## Results

A total of 24 patients who underwent tracheostomy for COVID-19-related ARDS were identified, with 13 in the ECMO group and 11 in the non-ECMO group. Patient baseline characteristics are reported in Table 1. There was no significant difference in race or gender between the groups (p>0.05). The ECMO group trended towards being younger with a mean age of 49 years vs. 59 years in the non-ECMO group, although this difference did not reach statistical significance (p= 0.064). Nine patients (69.2%) in the ECMO group had a history of hypertension compared to one patient (9.1%) in the non-ECMO group (p= 0.005). Otherwise, no significant differences were found in terms of patient comorbidities including obesity, cardiopulmonary, or renal disease. 

**Table 1:** Patient demographic characteristics by ECMO status Data are presented as median (IQR) for continuous measures, and n (%) for categorical measures ECMO: extracorporeal membrane oxygenation; IQR: interquartile range

	Total (N=24)	Non-ECMO (N=11)	ECMO (N=13)	p-value
Age at time of tracheostomy, median (IQR)	54.00 (44.00-60.50)	59.00 (47.00-65.00)	49.00 (40.00-58.00)	0.064
Gender, n (%)	-	-	-	0.095
Male	14 (58.33)	4 (36.36)	10 (76.92)	-
Female	10 (41.67)	7 (63.64)	3 (23.08)	-
Race, n (%)	-	-	-	0.93
Caucasian	10 (41.67)	4 (36.36)	6 (46.15)	-
Black	6 (25.00)	3 (27.27)	3 (23.08)	-
Hispanic	7 (29.17)	3 (27.27)	4 (30.77)	-
Other	1 (4.17)	1 (9.09)	0 (0.00)	-
BMI, median (IQR)	33.50 (30.50-40.50)	36.00 (30.00-42.00)	33.00 (31.00-39.00)	0.49
Diabetes, n (%)	-	-	-	0.44
No	11 (45.83)	4 (36.36)	7 (53.85)	-
Yes	13 (54.17)	7 (63.64)	6 (46.15)	-
Lung disease, n (%)	-	-	-	0.60
No	20 (83.33)	10 (90.91)	10 (76.92)	-
Yes	4 (16.67)	1 (9.09)	3 (23.08)	-
Hypertension, n (%)	-	-	-	0.005
No	10 (41.67)	1 (9.09)	9 (69.23)	-
Yes	14 (58.33)	10 (90.91)	4 (30.77)	-
Cardiovascular disease, n (%)	-	-	-	0.36
No	18 (75.00)	7 (63.64)	11 (84.62)	-
Yes	6 (25.00)	4 (36.36)	2 (15.38)	-
Chronic Kidney Disease, n (%)	-	-	-	1.00
No	19 (79.17)	9 (81.82)	10 (76.92)	-
Yes	5 (20.83)	2 (18.18)	3 (23.08)	-
Multiple intubations, n (%)	-	-	-	0.033
No	16 (66.67)	10 (90.91)	6 (46.15)	-
Yes	8 (33.33)	1 (9.09)	7 (53.85)	-

Complications and outcomes between the two groups are reported in Table 2. There was no significant difference in the overall incidence of tracheostomy complications between ECMO and non-ECMO groups (61.5% (n=8) vs. 27.3% (n=3), p= 0.12). Three of the ECMO patients underwent lung transplantation (23.08%, n=3), while none of the non-ECMO patients required lung transplant though this difference did not reach significance (p=0.22). Compared to non-ECMO patients, patients on ECMO had increased rates of overall postoperative bleeding (61.5% (n=8) vs. 18.2% (n=2), p= 0.047) and a higher mortality rate (38.5% (n=5) vs. 0.0% (n=0), p= 0.041). 

**Table 2:** Patient outcomes by ECMO status Data are presented as n (%) ECMO: extracorporeal membrane oxygenation

	Total (N=24), n (%)	Non-ECMO (N=11), n (%)	ECMO (N=13), n (%)	p-value
Lung transplant	-	-	-	0.22
No	21 (87.50)	11 (100.00)	10 (76.92)	-
Yes	3 (12.50)	0 (0.00)	3 (23.08)	-
Tracheostomy complications	-	-	-	0.12
No	13 (54.17)	8 (72.73)	5 (38.46)	-
Yes	11 (45.83)	3 (27.27)	8 (61.54)	-
Post-operative bleeding	-	-	-	0.047
No	14 (58.33)	9 (81.82)	5 (38.46)	-
Yes	10 (41.67)	2 (18.18)	8 (61.54)	-
Death	-	-	-	0.041
No	19 (79.17)	11 (100.00)	8 (61.54)	-
Yes	5 (20.83)	0 (0.00)	5 (38.46)	-

Among all patients, the average time from presentation to ICU admission was 3.5 days (IQR: 1.5-6.0) and average time from admission to tracheostomy was 27.50 days (IQR: 20.50-34.50) (Table 3). Patients placed on ECMO had a longer period from admission to intubation (seven days vs. three days, p= 0.002) and were more likely to have undergone multiple intubations (54% (n=7) vs 9% (n=1), p= 0.033). Patients in the ECMO group were cannulated for ECMO an average of 14 days (IQR: 11.0-22.0) prior to tracheostomy. No difference was found between ECMO and non-ECMO patients with regards to mean time from admission to tracheostomy placement (34 days vs. 24 days, p= 0.15) or time from intubation to tracheostomy placement (24 days vs. 21 days, p=0.66). Patients who weaned off ventilatory support did so at a median of 10 days if they were not on ECMO and at 57.5 days if they were on ECMO (p=0.16). ECMO decannulation occurred at an average of 19 days (IQR: 11.0-37.0) after tracheostomy placement. Patients who had their tracheostomy tubes decannulated achieved this within 78 days if they were not on ECMO and 146.5 days if they had required ECMO support (p=0.16). Mean hospital LOS was significantly longer for the ECMO group compared to the non-ECMO group (84 days vs 37 days, p<0.001).

**Table 3:** Different times of interest by ECMO status Time was measured in days for all variables; Data are presented as median (IQR). ECMO: extracorporeal membrane oxygenation; IQR: interquartile range; LATC: long-term acute care

	Total (N=24), median (IQR)	Non-ECMO (N=11), median (IQR)	ECMO (N=13), median (IQR)	p-value
Time from admission to ICU	3.50 (1.50-6.00)	3.00 (1.00-5.00)	5.00 (2.00-7.00)	0.18
Time from admission to intubation	6.00 (3.00-7.50)	3.00 (3.00-5.00)	7.00 (6.00-11.00)	0.002
Time from admission to ECMO	13.00 (12.00-15.00)	-	13.00 (12.00-15.00)	-
Time from admission to tracheostomy	27.50 (20.50-34.50)	24.00 (17.00-30.00)	34.00 (23.00-36.00)	0.15
Time from ECMO to tracheostomy	14.00 (11.00-22.00)	-	14.00 (11.00-22.00)	-
Total days intubated prior to tracheostomy	21.00 (15.00-26.00)	21.00 (14.00-25.00)	24.00 (15.00-27.00)	0.66
Time from tracheostomy until weaned off vent	45.00 (24.00-70.00)	10.00 (10.00-10.00)	57.50 (34.50-103.50)	0.16
Time from admission until ECMO decannulation	54.00 (38.00-70.00)	-	54.00 (38.00-70.00)	-
Time from tracheostomy until ECMO decannulation	19.00 (11.00-37.00)	-	19.00 (11.00-37.00)	-
Time from tracheostomy to tracheostomy decannulation	102.50 (55.00-149.00)	78.00 (51.50-102.50)	146.50 (76.00-158.00)	0.16
Time from admission until LTAC/rehabilitation	49.00 (33.00-82.00)	37.00 (29.00-45.00)	84.00 (71.50-138.00)	<0.001
Time from admission to death	45.00 (45.00-82.00)	-	45.00 (45.00-82.00)	-
Time from tracheostomy to LTAC/rehabilitation	23.00 (9.00-46.00)	10.00 (6.00-18.00)	48.50 (35.50-111.00)	<0.001
Time from tracheostomy to death	11.00 (7.00-59.00)	-	11.00 (7.00-59.00)	-
Time from ECMO to Death	30.00 (21.00-70.00)	-	30.00 (21.00-70.00)	-

## Discussion

Since the inception of the global COVID-19 pandemic, several clinical studies have aimed to elucidate the optimal timing and protocols for performing supportive measures such as tracheostomy and ECMO cannulation in patients with COVID-related ARDS. While nine-month mortality of patients requiring tracheostomy during hospitalization for COVID-19 has been reported to be up to 70%, recent data suggest that those undergoing tracheostomy after intubation for COVID-related ARDS have decreased mortality compared to intubated patients who did not receive a tracheostomy [18,19]. Furthermore, much of the published literature suggests better outcomes associated with early tracheostomy, defined as tracheostomy performed within 7-14 days of endotracheal intubation [2,3,7,16].

A large multinational cohort study evaluated the outcomes of 549 patients from 13 hospitals in four countries and found that while early tracheostomy (defined as within 14 days of intubation) had reduced ventilator dependence and LOS, it also lowered 30-day risk-adjusted survival [20]. It is also important to note, however, that robust evidence exists in support of the contrary. A large systematic review, meta-analysis, and meta-regression by Battaglini et al. demonstrated no association between the timing of tracheostomy and COVID-19-related outcomes [5]. For those on ECMO support, tracheostomy has been reported to be performed safely and at rates similar to practices in pre-COVID-19 viral pneumonia [21-23]. However, it remains unclear whether tracheostomy should be performed while on ECMO or if the decision to perform tracheostomy should be delayed until after ECMO has been weaned. An analysis by Kohne et al. showed similar mortality between those who did and did not receive a tracheostomy, with placement of a tracheostomy being associated with increased patient mobilization [22]. Furthermore, Smith et al. published a case series of 37 patients undergoing tracheostomy while on ECMO. Their population experienced minimal adverse events [21]. On the contrary, a large international, multi-center, retrospective study on tracheostomy management in patients on ECMO suggested that tracheostomy during ECMO was not associated with improved sedation, analgesia, or level of consciousness [23]. The authors suggest that this, coupled with an increased risk of post-procedural bleeding, encourages reconsideration of tracheostomy in these patients. In addition, Matsuyoshi et al. recommended performing tracheostomy after weaning from ECMO, in part due to the increased risk of hemorrhagic complications [17]. As such, the optimal protocol for performing tracheostomy on patients requiring ECMO for COVID-related ARDS remains in question. 

More recently, Staibano et al. published a case series of 24 patients with COVID-19 who underwent tracheostomy while on ECMO. The authors collected several data points paralleling those of our study, including a mean of 26 days from intubation to tracheostomy, compared to 24 days in our ECMO population, 20 days from ECMO cannulation to tracheostomy versus 14 days in our ECMO population, and a 33% mortality rate, compared with 38% in our ECMO population. Their population had a total length of stay of 119 days versus 84 days in our ECMO group, though there are surely a multitude of confounding factors contributing to this [24]. Another noteworthy recent study, which is one that most closely resembles ours, is that by Son et al. [25]. They performed a retrospective study investigating outcomes of percutaneous dilatational tracheostomy in patients with COVID-19 supported by ECMO (n=17) compared to those without ECMO (n=12). Similar to our study, they noted no significant difference in gender, BMI, or time from intubation to tracheostomy (17 vs. 18 days, p=.477), and a higher rate of bleeding in the ECMO group (59% vs 0%, p=.001). Their ECMO group had a mean time from ECMO cannulation to tracheostomy of 14 days, identical to our ECMO group, and a mean time from tracheostomy to ECMO decannulation of 15 days compared to 19 days in our population. They, too, noted that their ECMO group was nearly 10 years younger than the non-ECMO group, although their difference did reach statistical significance (56 vs. 68 years, p=.035). Interestingly, their population experienced a higher overall mortality rate without a significant difference between the two groups (53% vs. 58%, p=.792) [25]. 

Our study demonstrates that ECMO cannulation prior to tracheostomy for COVID-related ARDS is associated with poorer outcomes. Principally, our patient population undergoing tracheostomy while on VV-ECMO had a significantly higher mortality rate and endured significantly longer hospital stays than those not on ECMO. This may indicate a more severe disease burden in this population. Perhaps this is due to the inferior baseline health status of the ECMO group given its higher prevalence of hypertension; however, the two groups were otherwise not significantly different in terms of age, gender, race, or other comorbidities. While there was no difference in the length of intubation prior to tracheostomy, the ECMO group did have a significantly longer time from hospital admission to intubation. This may suggest that delays in intubating patients early in their hospitalization ultimately led to a more severe disease course that resulted in these patients requiring ECMO cannulation. Early in the pandemic, with limited data and understanding of COVID-19, there was initial hesitation to intubate patients early due to concerns for viral transmission to healthcare workers and potential increased mortality for patients. A recent meta-analysis of 20 observational studies sought to evaluate the outcomes of early vs. late intubation in COVID-19-related ARDS patients and determined that early intubation within 24-48 hours of ICU admission showed 119 fewer deaths per 1000 patients, reduced LOS by 2.81 days, and decreased length of ventilatory support by 2.12 days [26].

With regards to post-procedural bleeding, our study is consistent with prior studies demonstrating an increased risk of hemorrhagic complications in patients undergoing tracheostomy while on ECMO, which is likely due to the requirement of in-line anticoagulation to prevent thrombotic complications [27]. 

Our retrospective, case-control study suggests that patients with COVID-related ARDS who underwent tracheostomy while on ECMO support had increased mortality and hemorrhagic complications compared with those not on ECMO. This may imply that, in the event of future respiratory viral epidemics, tracheostomy should be performed earlier in the intubation course, prior to initiation of ECMO, or following decannulation from ECMO. 

Our study is limited by its small sample size, raising the possibility of Type II errors. Our data demonstrated several considerable differences between the two groups that did not reach statistical significance, likely due to low power. Examples of this include a mean age difference of 49 vs. 59 years old, median time to weaning from ventilatory support of 10 days in the non-ECMO group vs. 57.5 days in the ECMO group, and mean time to decannulation of 78 days in the non-ECMO group vs. 146.5 days in the ECMO group. Further, disease severity may be a major confounder for the difference in outcomes between the ECMO and non-ECMO groups. Finally, the COVID-19 pandemic remained dynamic throughout the study period as different COVID variants brought varying degrees of virulence, and clinical practices were constantly evolving. This likely led to intrinsic variations in the study population’s disease severity and how patients were managed.

## Conclusions

Patients who underwent ECMO cannulation prior to tracheostomy for COVID-19-related ARDS had poorer outcomes. These patients had a high mortality rate. They also suffered from significantly higher rates of postoperative hemorrhage compared with those who were not on ECMO. Further, these patients endured longer hospitalizations. It is unclear whether these poorer outcomes are related to a more severe disease burden in patients on ECMO. Additional investigation is needed to determine the ideal balance between ECMO and tracheostomy timing, with the goal of guiding the management of future respiratory viral epidemics.
